# Echocardiographic Diagnosis and Hemodynamic Evaluation of Patent Ductus Arteriosus in Extremely Low Gestational Age Newborn (ELGAN) Infants

**DOI:** 10.3389/fped.2020.573627

**Published:** 2020-11-19

**Authors:** Yogen Singh, Alain Fraisse, Omer Erdeve, Begum Atasay

**Affiliations:** ^1^Department of Pediatrics - Pediatric Cardiology and Neonatal Medicine, Cambridge University Hospitals NHS Foundation Trust, Cambridge, United Kingdom; ^2^University of Cambridge Clinical School of Medicine, Cambridge, United Kingdom; ^3^Pediatric Cardiology Services, Royal Brompton Hospital, London, United Kingdom; ^4^Imperial College London, National Heart and Lung Institute, London, United Kingdom; ^5^Division of Neonatology, Department of Pediatrics, Ankara University School of Medicine, Ankara, Turkey

**Keywords:** patent ductus arteriosus (PDA), extreme preterm infants, clinical decision making, echocardiographic evaluation of PDA, ELGAN extremely low gestational age newborn

## Abstract

Persistent Patent ductus arteriosus (PDA) is a common finding in extremely low gestational age newborn infants and its prevalence is inversely proportional to the gestational age. The presence of a persistent PDA is associated with increased mortality and several significant morbidities including intraventricular hemorrhage, pulmonary hemorrhage, necrotizing enterocolitis, and chronic lung disease or bronchopulmonary dysplasia. However, treating PDA has not been demonstrated to have beneficial impact on the long term outcomes. Currently there is no consensus on whether to treat the PDA or not, and if treat, when to treat and how to treat. The echocardiography is the investigation of choice to diagnose PDA, estimating the magnitude of shunt volume and assessing its hemodynamic significance, and to exclude/diagnose any associated congenital heart defect before any intervention. Various echocardiographic parameters and staging/scoring systems have been described to help the clincians making the clinical decisions and some of theses scoring systems are quite complex to apply in a busy day to day clinical practice. This concised review paper is focused to help the clinicians in making a clinical decision based upon clincial and echocardiography parameters. Hence, only the parameters which are commonly used and helpful in making the clinical decisions in day to day clincial practice have been described in this paper.

## Introduction

Patent ductus arteriosus (PDA) is an essential component of the fetal circulation and in most of the term infants it closes soon after birth. However, PDA is known to remain persistent in large proportion of extremely low gestational age newborn (ELGAN) infants and its prevalence is inversely proportional to the gestational age (1). The incidence of a persistent PDA in infants born <1,000 g or those under 28 weeks of gestation is around 66% (1, 2).

The important risk factors for persistent PDA include lower gestational age, lack of antenatal steroids, and need for mechanical ventilation. The risk of persistent PDA increases with decreasing gestational age (GA) and lower birth weight (3)^1^ (4). Closure is also less likely to occur in infants who have neonatal respiratory distress syndrome needing mechanical ventilation and in those who did not receive antenatal corticosteroids (4, 5).

The presence of a persistent PDA is associated with increased mortality and several significant morbidities including intraventricular hemorrhage (IVH), pulmonary hemorrhage, necrotizing enterocolitis (NEC), and chronic lung disease (CLD) or bronchopulmonary dysplasia (BPD) (6–8). Despite large number of trials, research studies and scientific efforts in making an evidence based consensus on how best to manage the PDA in this vulnerable group of patients, there has been no agreement on how best to treat or even how best to assess the PDA and its impact (9). There continues to be a clinical dilemma who should we treat, how to treat and what the best strategy in the ELGAN infants is. Different treatment strategies have been studied over the years. These include prophylactic treatment, early targeted treatment, treatment of a clinically symptomatic PDA, the conservative approach of “wait and watchful policy,” surgical ligation and recently described percutaneous transcatheter closure of PDA (9–12).

This review focuses at clinical decision making in managing PDA in the ELGAN infants. The aim of the paper is not to advice on the type of treatment, but primarily looking on the role of echocardiography in hemodynamic evaluation of PDA which can help in making clinical decision on the bedside.

## Clinical Diagnosis of PDA

**Figure 1:**
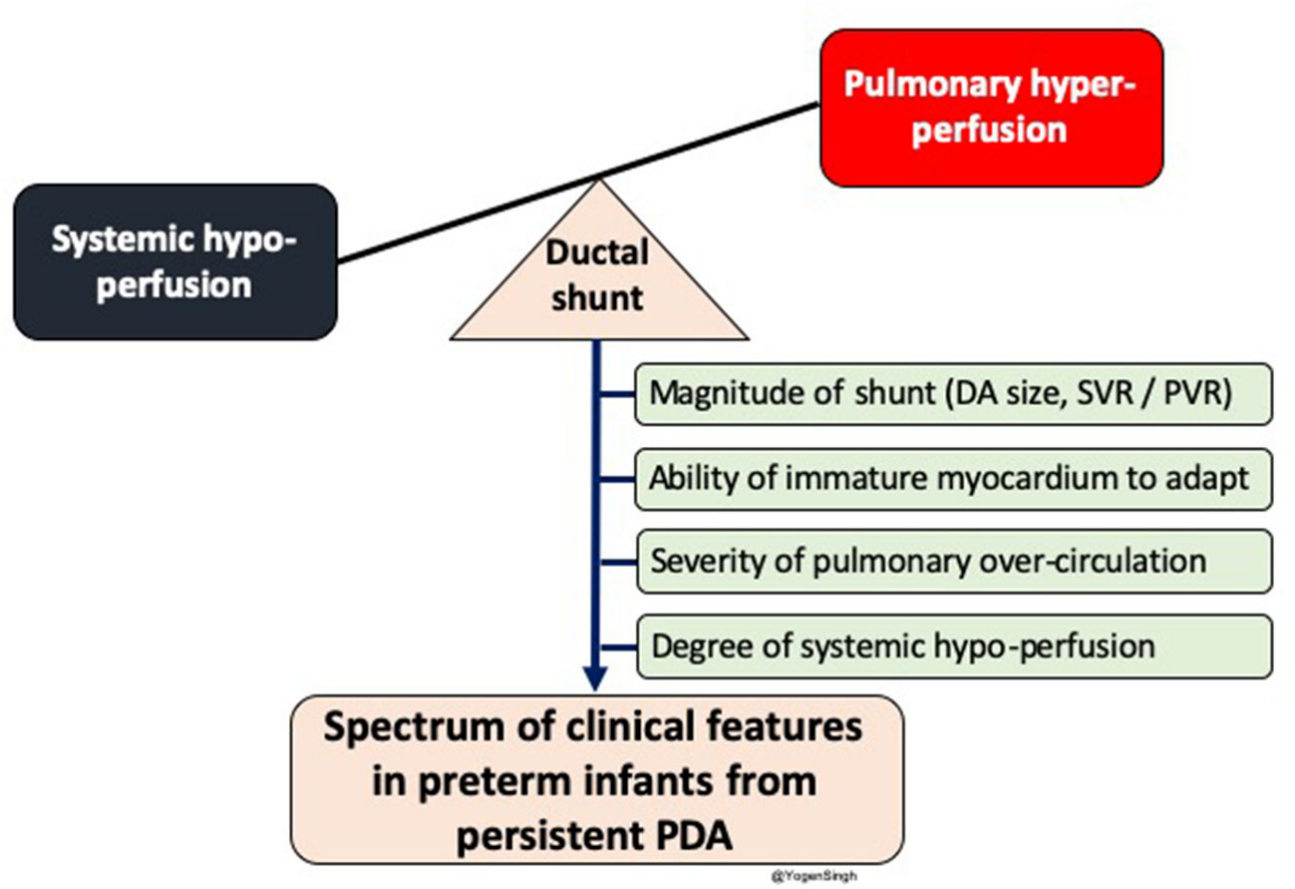
Diagram showing impact of significant left to right shunt across ductal arteriosus (DA) leading to pulmonary over-circulation and systemic hypoperfusion. Spectrum of clinical features in preterm infants depends upon magnitude of ductal shunt, which depends upon DA size and balance between systemic and pulmonary vascular resistance, and inability of immature myocardium to adpat to circulatory disturbance. PDA, patent ductus arteriosus; SVR, systemic vascular resistance; PVR, pulmonary vascular resistance.

The clinical signs of PDA depend upon amount of the shunt volume passing across the ductus arteriosus; which primarily depends upon the systemic and pulmonary vascular resistance, ability of the myocardium to adapt to increased shunt volume and size of the ductus arteriosus (13–15) (Figure 1).

Infants with even a large PDA often have no clinical signs in the first few days after birth because of persistently high pulmonary vascular resistance (PVR) leading to decreased amount of PDA shunt volume. As PVR drops, shunt volume increases and infant develops the signs and symptoms of PDA. Initially, heart murmur may be heard and often the infant is noted to have fleeting desaturations. As PDA persists then further signs such as hyperactive precordium, bounding pulses and widened pulse pressure are recognized and when myocardium fails to adapt to this increased shunt volume the signs of heart failure develop (15, 16). However, a small proprotion of ELGAN infants with decreased myocardial adaptability may develop hypotension and acidosis even during first few days (16).

Clinical factors and biomarkers can be used both to diagnose PDA and assess its hemodynamic significance In hemodynamically significant PDA (hsPDA), signs and symptoms of pulmonary overcirculation and systemic hypoperfusion are rated by looking at the oxygenation difficulty, number and severity of apnea and desaturations, need and extent of non-invasive or invasive respiratory support, feeding intolerance, radiologic evidence of cardiomegaly and pulmonary edema, presence of oliguria, low mean or diastolic hypotension with or without metabolic acidosis requiring cardiotropic or vassopressor drugs (17). However, most clinical signs have limited sensitivity in the first day of life and hence, there is a few days lag in clinical diagnosis of PDA as compared to echocardiography (15, 17).

Moreover, it is very difficult to diagnose or rule out underlying congenital heart defect on clincial examination alone (18). Hence, echocardiographic diagnosis is mandatory before any clincial intervention.

## Echocardiographic Evaluation of PDA: Diagnosis and Assessment of Hemodynamic Impact of Shunt Volume

**Figure 2:**
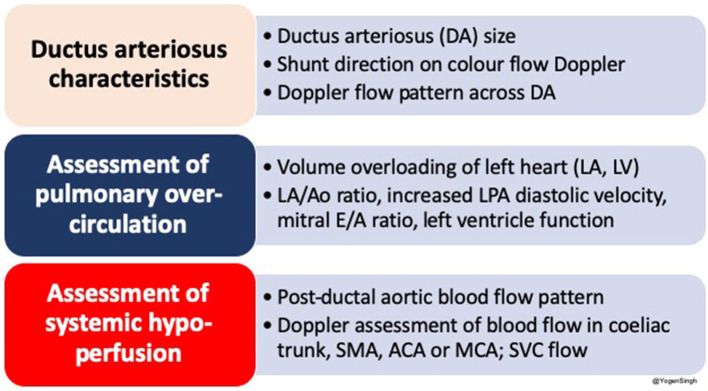
Summary of an approach to echocardiographic assessment of PDA and hemodynamic evaluation; LA, left atrium; LV, left ventricle; DA, ductus arteriosus; Ao, aorta; SMA, superior mesenteric artery; ACA, anterior cerebral artery; MCA, middle cerebral artery; SVC, superior vena cava.

The echocardiography is the gold standard bedside investigation to diagnose PDA. In addition to make a confirmative diagnosis of PDA and exclude/diagnose any associated congenital heart defect (CHD), it can help in estimating the magnitude of shunt volume and assessing its hemodynamic significance—it can be used to assess the hemodynamic impact from pulmonary overcirculation and systemic hypoperfusion due to shunt volume (13–15). This could be systematically achieved by studying: (a) ductal characteristics, (b) parameters of pulmonary overcirculation, and (c) signs of systemic hypoperfusion (Figure 2).

Various echocardiographic parameters have been described in the research setting and complex staging/scoring systems (17, 19). This review paper is focused to help the clinicians in making a clinical decision on the bedside and hence the parameters which are commonly used and helpful in day to day common clinical practice have been described.

### Echocardiographic Assessment of Ductal Characteristics

The echocardiography can be used to assess the size of PDA by measuring transductal diameter, interrogate shunt direction, and velocity of blood flow across the ductus arteriosus can be measured by using Doppler technique.

#### Measuring Transductal Diameter

**Figure 3:**
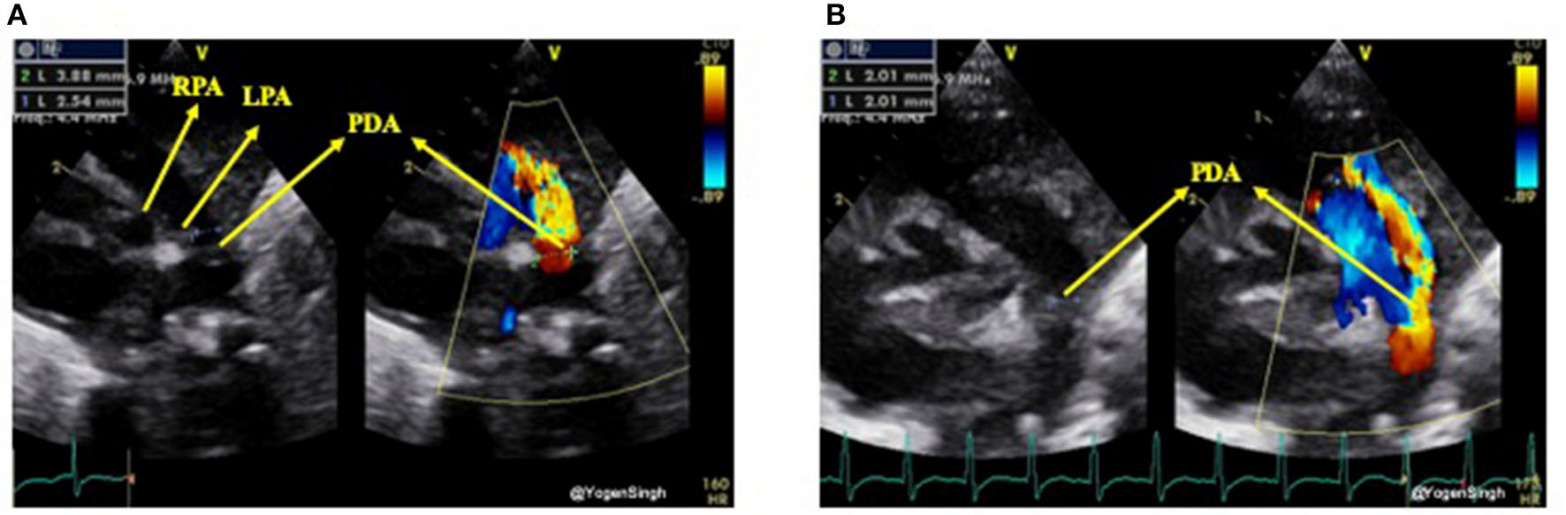
Measurement of ductal size on 2D and color Doppler on high left parasternal “ductal view.” **(A)** Showing significant discrepency between ductal diameter measurement on 2D and color Doppler—over-estimation of ductal size on color Doppler because of gain setting; **(B)** showing no significant discrepency between ductal diameter measurement on 2D and color Dopple after optimisation of gain setting. LPA, left pulmonary artery; RPA, right pulmonary artery; PDA, patent ductus arteriosus.

Although the PDA can be visualized from many windows, the high left-sided parasternal “ductal” view is the preferred window to obtain a clear 2D image and accurately measure size of the ductus arteriosus. PDA size is measured from the transductal diameter at the site of maximum constriction (narrowest dimension), which is usually at the pulmonary end (20). Most studies have described measuring PDA size using color Doppler, although with new ultrasound machines and through proper training it can be easily measured on 2D image. If color Doppler is used to measure the duct size the gain setting should be adequately optimized to minimize the risk of over-estimation. Color compare or simultaneous mode, which allows putting 2D and color Doppler image side by side, can be applied to measure ductal size in both modes using frame by frame technique (Figure 3).

#### Direction of Shunt Across Ductus Arteriosus

**Figure 4:**
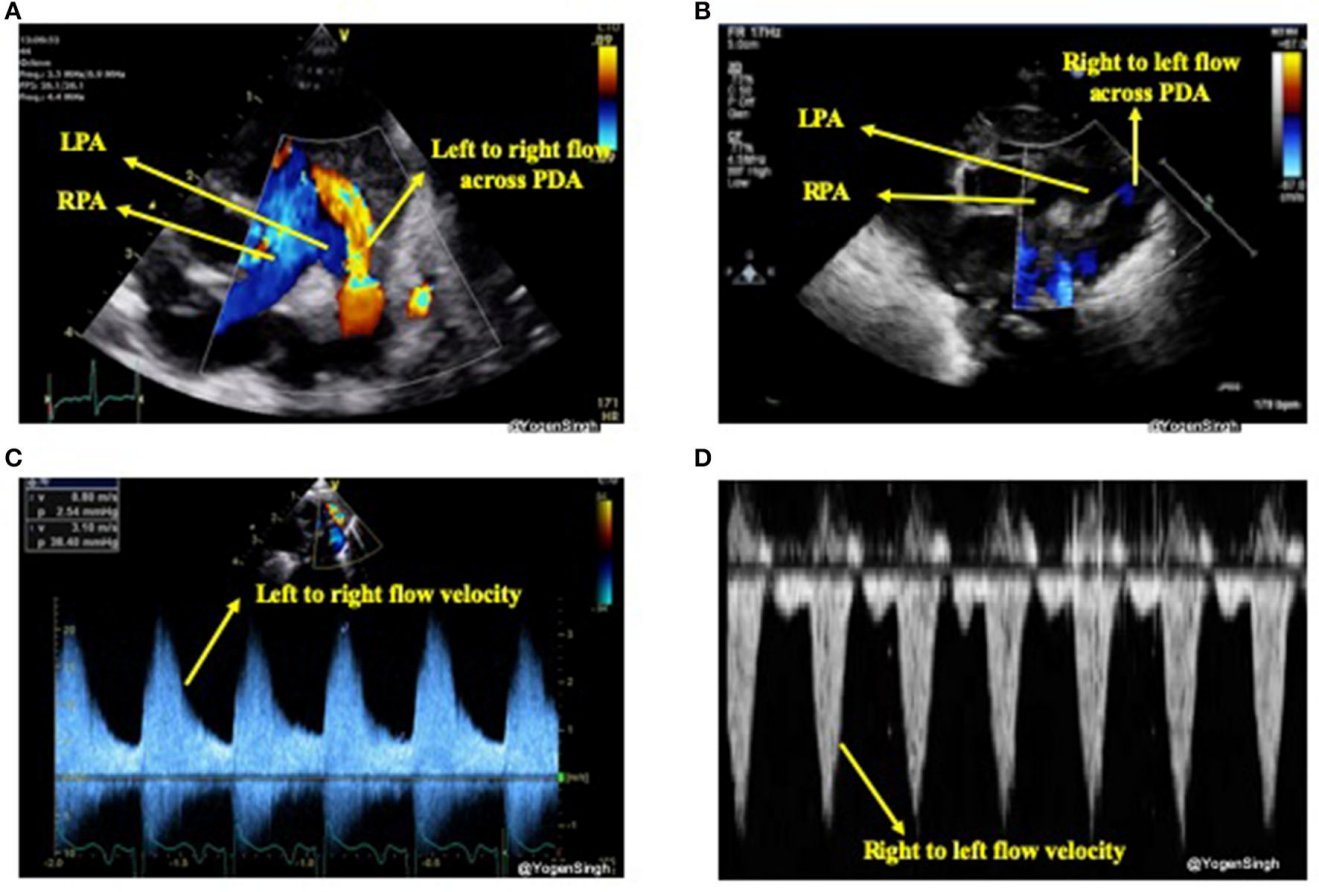
Assessment of PDA shunt direction on color flow and with Doppler application. **(A)** Left to right shunt seen as red (blood coming toward probe) while blood in branch pulmonary arteries seen as blue (blood going away from probe); **(B)** showing right to left shunt seen as blue color—similar to branch pulmonary arteries in a view “three legged trouser;” **(C)** Doppler assessment showing left to right shunt (above the baseline as blood coming toward the probe); and **(D)** Doppler assessment showing right to left shunt (below the baseline as blood going away from the probe). LPA, left pulmonary artery; RPA, right pulmonary artery; PDA, patent ductus arteriosus.

The direction of the ductal shunt depends upon the relationship between the pulmonary and systemic pressures. It is assessed using color Doppler and direction of blood flow across ductus arteriosus is normally left to right, from the aorta (high systemic pressure) to the pulmonary artery (low pulmonary pressure) but it can be right to left or bi-directional when there is high pulmonary vascular resistance or when there is anatomical cause (due to certain CHDs). With conventional setting left to right shunt is seen as red jet while right to left shunt is seen as blue (21). A right-to-left shunt across the PDA is more difficult to see because color Doppler will show it as a blue jet, blood going toward aorta from pulmonary end, similar to branch pulmonary arteries. Color compare or simultaneous mode can be very helpful in such situation. Bi-directional flow is often seen during transitional circulation or when the pulmonary artery pressures are equal to systemic pressures (21). Shunt direction can also be assessed using pulse or continuous wave Doppler where left to right shunt is seen above the baseline (blood coming toward the probe) while right to left shunt is seen below the baseline (blood going away from the probe) (15, 21) (Figure 4).

#### Velocity of Shunt Across PDA and Its Significance

**Figure 5:**
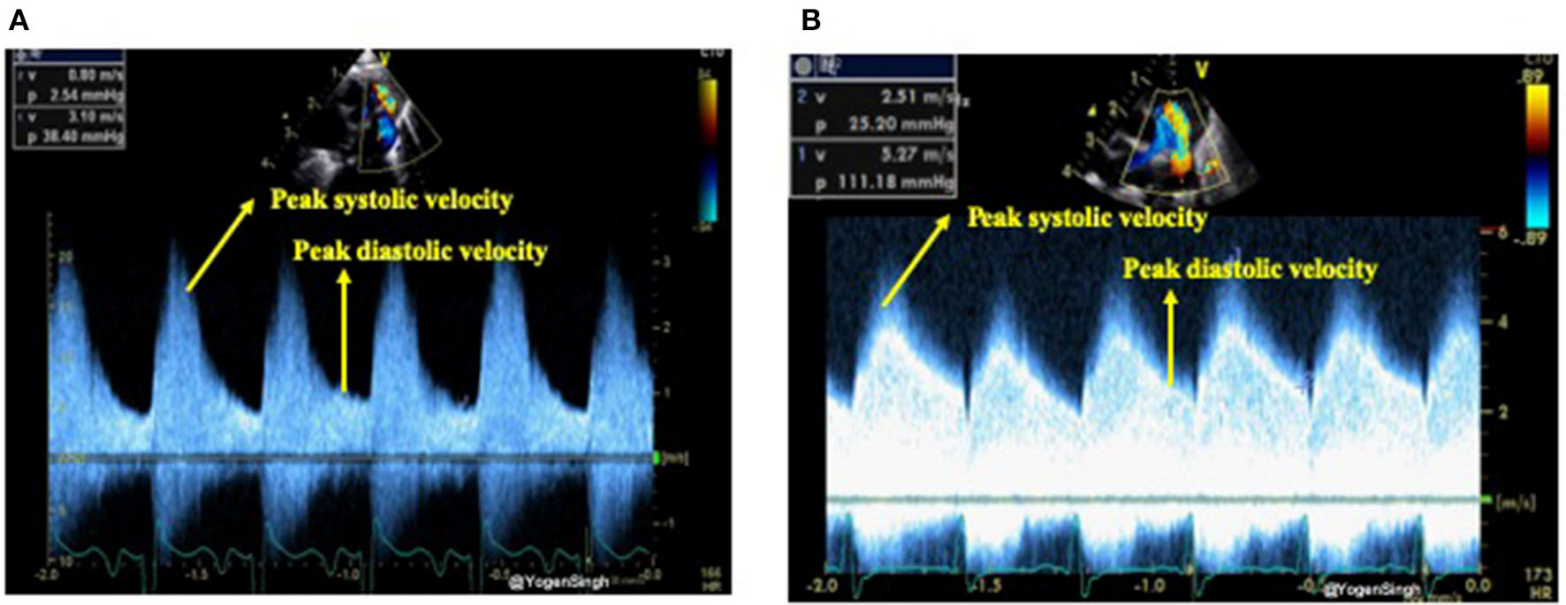
Assessment of restrictive and non-restrictive (pulsatile) flow pattern on Doppler assessment of PDA. **(A)** Showing non-restrictive (pulsatile) flow pattern with end-diastolic velocity (EDV) less than half of the peak systolic velocity; and **(B)** showing restrictive flow pattern with end-diastolic velocity (EDV) more than half of the peak systolic velocity.

The shunt velocity across the PDA during the cardiac cycle can be obtained by applying pulse or continuous wave Doppler in the ductus arteriosus. The maximum velocity during systole and diastole can be measured. Non-restrictive shunts have a low peak systolic velocity with a high systolic to end-diastolic velocity gradient while restrictive shunts have a high peak systolic velocity and a low systolic to diastolic velocity gradient. If the ratio between peak systolic and end-diastolic velocity is >2 then it is considered as a pulsatile flow pattern while a ratio of <2 is described as restrictive shunt suggestive of a closing PDA (22, 23) (Figure 5).

### Echocardiographic Evaluation of Pulmonary Overcirculation

**Figure 6:**
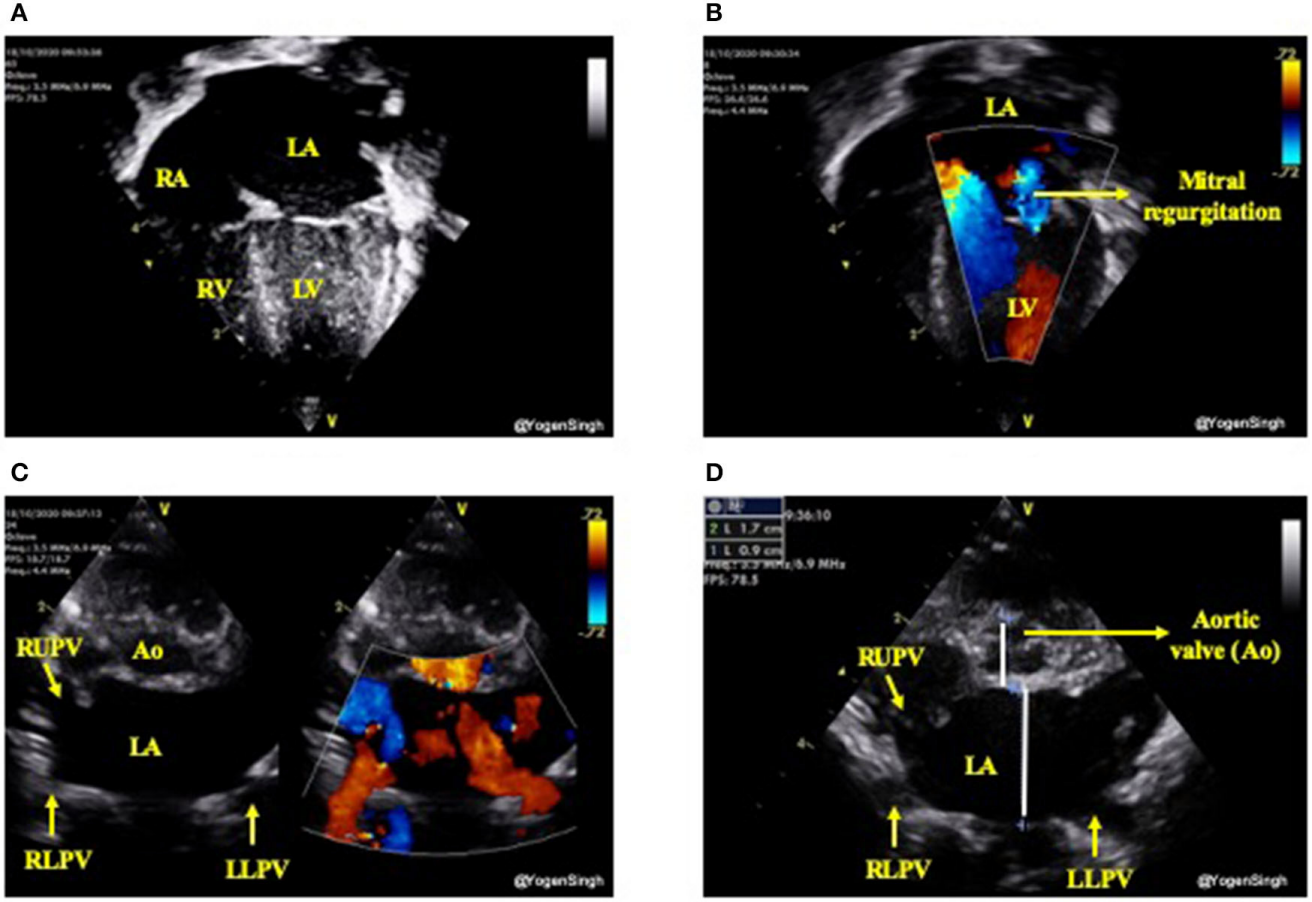
Assessment of left heart volume overloading on visual inspection “eyeballing.” **(A)** Apical 4 chamber view in 2D showing dilated left side of the heart (dilated left atrium and left ventricle); **(B)** Mitral regurgitation on color flow mapping as blue jet going back to left atrium (see explanation in text); **(C)** “Crab view” showing dilated pulmonary veins reflecting increased pulmonary venous return and **(D)** Dilated left atrium in parasternal short axis view—on visual inspection LA looks almost the double the size of aortic valve (Ao). LA, left atrium; LV, left ventricle; RA, right atrium; RV, right ventricle; Ao, aortic valve; RUPV, right upper pulmonary vein; RLPV, right lower pulmonary vein; LLPV, left lower upper pulmonary vein.

The increased pulmonary blood flow from a significant left-to-right ductal shunt leads to pulmonary blood flow and hence increased pulmonary venous return. This leads to increased volume overload in the left atrium (LA) which gradually gets dilated and if this process of significant left to right shunt persists then it leads to dilatation of left ventricle from increased preload, especially in absence of a large intra-atrial shunt. As aortic valve annulus (Ao) is a relatively fixed structure and it does not get dilated due to left heart overloading. Hence, a ratio of LA/Ao can be used as a surrogate of increased pulmonary venous return (24, 25). Similarly, the left ventricular end diastolic diameter (LVEDD) can be used as surrogate markers for pulmonary venous return. In clinical practice the volume overloading of the left heart can be subjectively assessed by “eyeballing” (15) (Figure 6).

**Figure 7:**
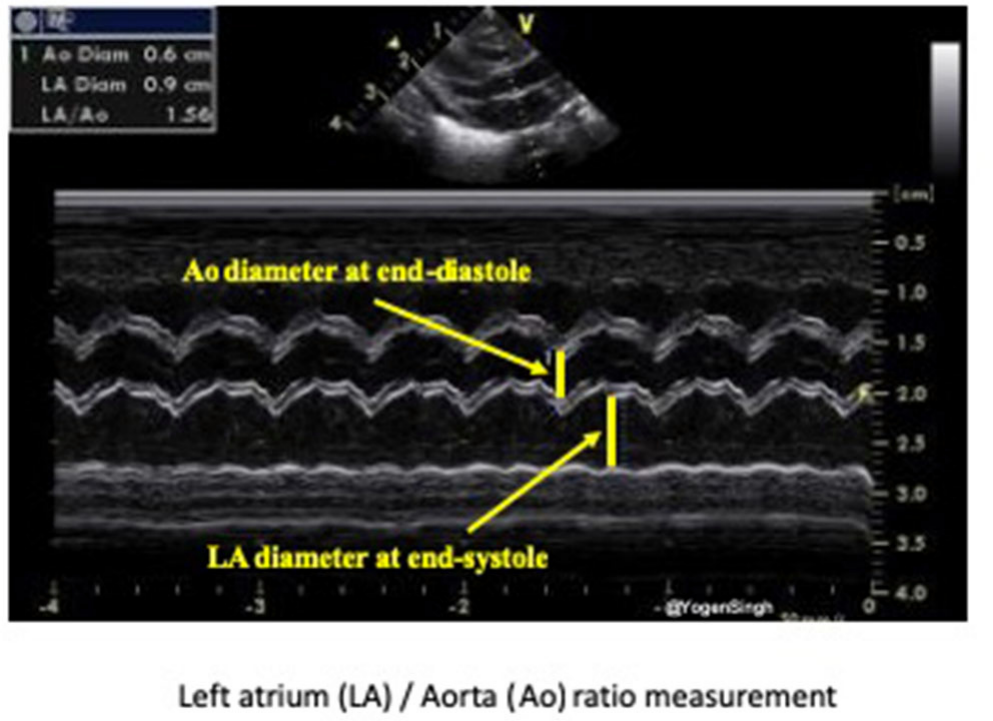
Assessment of left atrium (LA) to aorta (Ao) ratio in parasternal long axis view. LA and Ao diameter measurement shown using M-mode and cartoon schematic diagram.

Both LA/Ao ratio and LVEDD can be measured from the parasternal long axis view using M-mode with the cursor perpendicular to the aorta at the level of the aortic valve or at the septum at the tip of the mitral valve leaflets, respectively (Figure 7). LA/Ao ratio of >1.4 is considered significant and has been used as a cut-off value in many clinical trials (25). The normal reference ranges for LVEDD in preterm infants in relation to body weight and postnatal age have been published and *z*-scores should be used for LVEDD (26).

Variable degree of mitral valve insufficiency is often seen in infants with persistent large PDA and significant left heart dilatation (Figure 6). It occurs due to left atrial dilatation resulting in stretching of mitral valve and left ventricular volume-overloading. The mitral valve regurgitation usually improves significantly with normalization of left atrial size and resolves completely within weeks after PDA closure (14, 26, 27).

While assessing left heart volume overloading one should be mindful of intra-atrial shunt. A large left-to-right shunt through the foramen ovale or septal defect can “offload” the left side of the heart even in the presence of a significant ductal shunt leading to an artificially low/normal LA/Ao ratio or low LVEDD.

**Figure 8:**
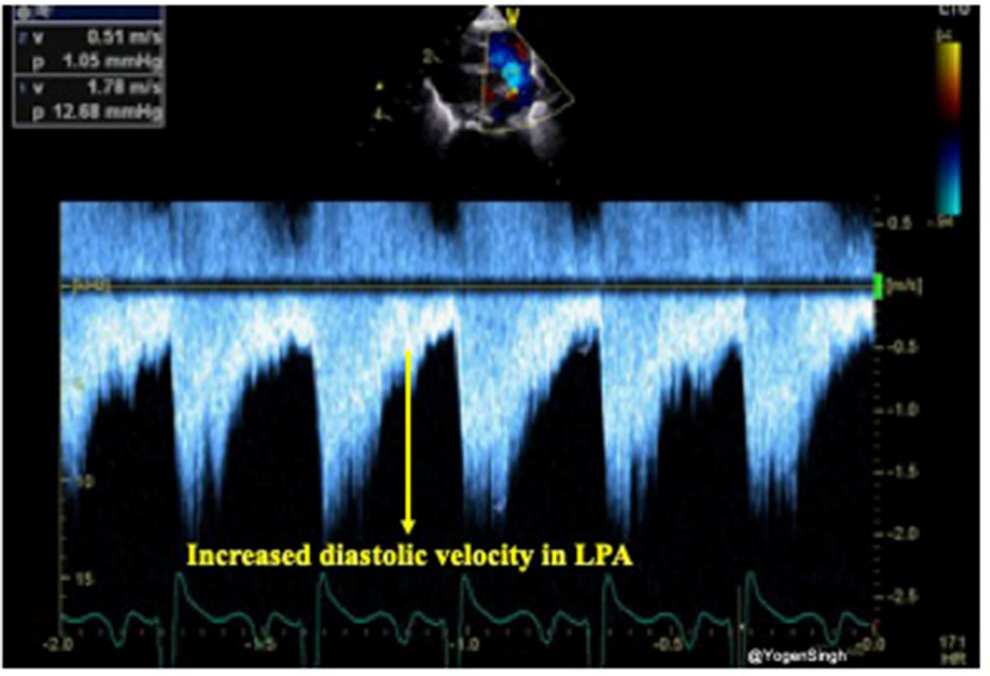
Doppler assessment of blood flow in left pulmonary artery (LPA) showing increased diastolic velocity indicative of significant ductal shunt in diastole leading to turbulence and increased velocity.

The presence of forward pulmonary flow in diastole in the left pulmonary artery (LPA) has been described as a sign of significant left to right shunt through the PDA. Using pulsed wave Doppler in the LPA mean and end-diastolic velocity can be measured and cut-off points of 0.42 and 0.20 m/s, respectively, have been described as indicative of significant ductal shunt (24) (Figure 8).

The mitral valve E/A ratio refers to the ratio of the velocity of the early (E) diastolic phase of ventricular filling vs. the late atrial (A) contraction component. Mitral valve E/A ratio can be obtained from apical 4-chamber view with the pulsed Doppler range gate set slightly below the mitral valve annulus. In preterm infants, mitral valve E/A ratio is usually <1 due to poor compliance of the myocardium leading to moderate impairment of diastolic performance and low early diastolic filling velocity. In the presence of a hsPDA, atrial pressure increases because high pulmonary venous return and this leads to a reversal of the E/A ratio >1. Various other echocardiographic parameters have been studied and described to assess pulmonary circulation such as left ventricular output (LVO) to superior vena cava flow (SVC) ratio, and decreased isovolumic relaxation time (IVRT) using tissue Doppler Imaging (TDI) (13, 24, 28). There may be limited expertise in doing TDI assessment accurately on neonatologist performed echocardiography. On the other hand, echocardiographic assessments (such as SVC flow and LVO estimation) needing multiple parameters are not only time consuming but also has potential to make errors in measurement and significant intra- and inter-observer variability (29–32). Hence, the common echocardiographic parameters often used in clinical decision making on the bedside remain qualitative assessment on visual inspection “eyeballing,” LA/Ao ratio and LVEDD measurement. Mitral E/A ratio is easy to measure but one should be mindful that even in preterm infants with no hsPDA E/A ratio gradually become >1 with time as myocardium compliance improves.

### Echocardiographic Evaluation of Systemic Hypoperfusion

**Figure 9:**
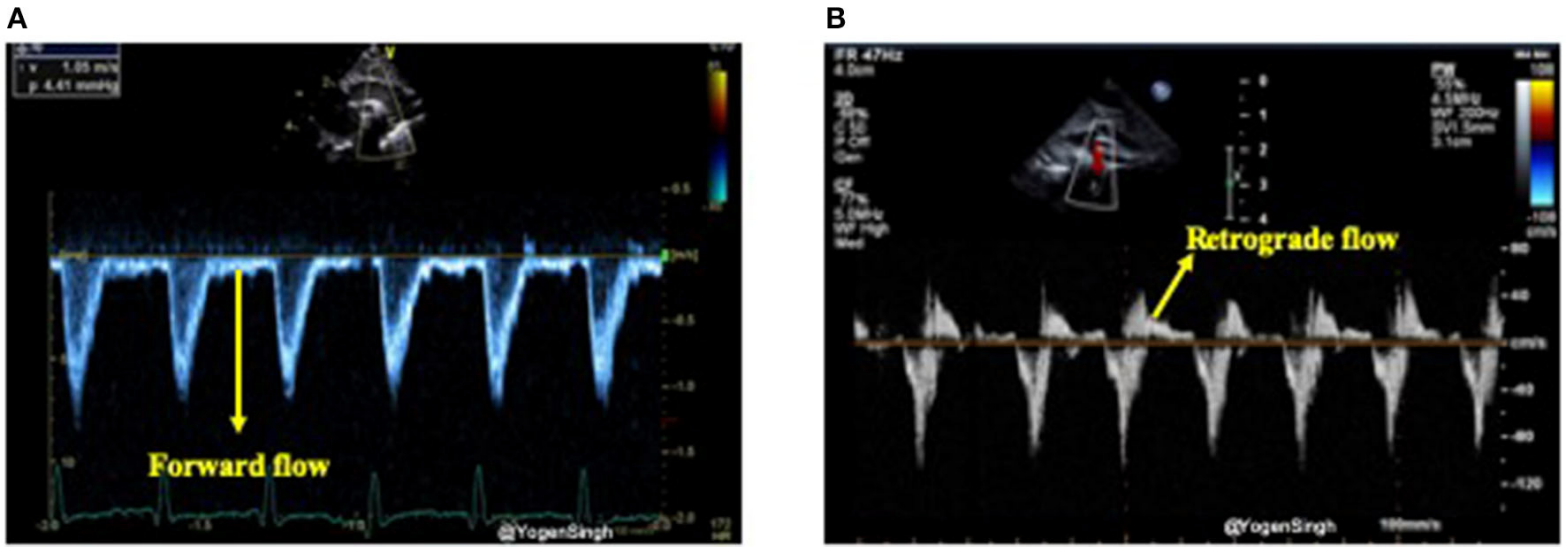
Doppler assessment of blood flow in descending aorta (post-ductal flow). **(A)** Showing forward blood flow during disatole; and **(B)** showing retrograde blood flow during diastole indicating “ductal steal” in presence of a large PDA.

In the presence of a large PDA, blood shunts away from the systemic circulation throughout the cardiac cycle, however, this becomes more apparent during diastole and it can be studied using Doppler on echocardiography (15, 31). Retrograde or absent blood flow during diastole in descending aorta below the ductal ampulla or in the coeliac axis or superior mesenteric artery have been described as indicator of significant PDA shunt leading to systemic steal (systemic hypoperfusion) (24, 32). Doppler flow patterns from the descending aorta can be obtained from a suprasternal or high parasternal view with the pulsed wave Doppler sample gate placed distal to the origin of ductus arteriosus (ductal ampulla) (Figure 9).

**Figure 10:**
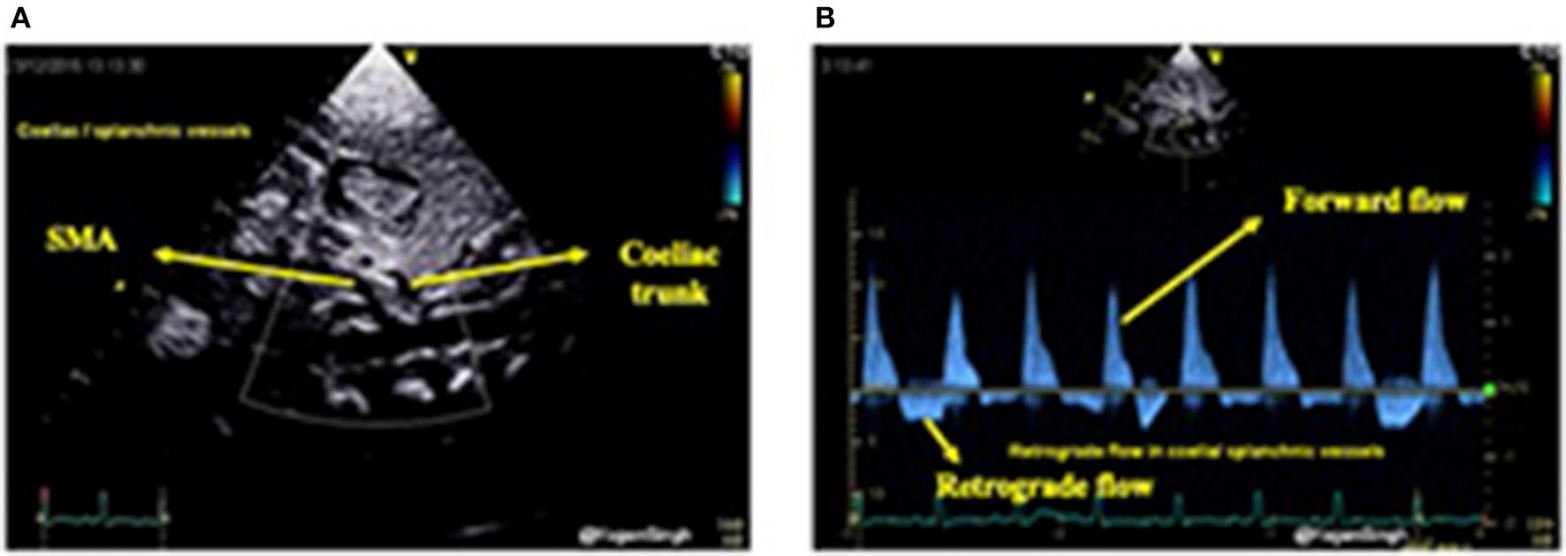
Color flow mapping and Doppler assessment of blood flow in the coeliac trunk and superior mesenteric artery in sub-costal sagittal view. **(A)** Showing color flow mapping of the coeliac and superior mesenteric arteries; and **(B)** showing retrograde blood flow during diastole in the the coeliac and superior mesenteric arteries indicating “ductal steal” in presence of a large PDA.

Similarly, celiac trunk or superior mesenteric artery can be interrogated using pulsed wave Doppler in the sagittal abdominal view (Figure 10). Doppler assessment of the anterior cerebral artery in the mid-sagittal view of brain ultrasound can be performed and retrograde flow during diastole would suggest significant ductal shunt—similar to coeliac or superior mesentery artery Doppler assessment. However, to date the clinical relevance and long term outcomes of the deranged cerebral Doppler flow patterns remain unknown (33).

Based upon the clinical and echocardiographic criteria various staging systems been described and have been shown to help in decision making for intervention (17, 19). However, in authors experience they have not been widely adopted in the clinical practice which could be possibly because of their extensive number of parameters. Recently, van Laere et al. (13) suggested essential various echocardiographic parameters including measuring left ventricular output in all infants needing assessment of PDA. However, in authors experience these staging systems needing extensive echocardiographic parameters assessment have not been widely adopted in the clinical practice. We have summarized the commonly used parameters which would help the clinicians assessing the PDA and its hemodynamic significance on echocardiography, and in making clinical decisions in the clinical practice.

## Clinical Decision Making: is the PDA Hemodynamically Significant?

The above echocardiographic parameters summarized in Table 1 may help in addressing the issue of whether the PDA is hemodynamically significant or not. However, the definition of an hsPDA continues to evolve. In addition to multiple echocardiographic indices described above, the hemodynamic significance of a persistent PDA should be interpreted by considering the gestational and chronological age, and by assessing the vulnerability of organs at risk for overflow (the lungs), or hypoperfusion (e.g., the brain, intestines, and kidneys). Further work is needed to reach a consensus on how to define and manage a hemodynamically significant persistent PDA in extremely preterm infants (34).

**Table 1:** Summary of essential parameters used for echocardiographic assessment and hemodynamic evaluation of PDA in ELGAN infants.

**PDA evaluation criteria**	**Essential echocardiographic parameters for assessment of PDA and hemodynamic evaluation**
Ductal characteristics	• PDA size (small <1.5 mm, moderate 1.51–2 mm, large >2 mm) and flow direction (left to right, right to left, or bi-directional) and Doppler assessment with maximum velocity (Vmax) in systole and end-diastole
Assessment of pulmonary over-circulation	• Dilated left side of the heart on visual inspection “eyeballing” and LA/Ao ratio (mild <1.4, moderate 1.41–1.6, severe >1.6) OR LVEDD (correlate with *z*-scores) OR LPA diastolic velocity, mean velocity >0.42 m/s, end-diastolic velocity >0.2 m/s OR Reversal of mitral E/A ratio *Document presence or absence and magnitude of intra-atrial shunt
Assessment of systemic hypoperfusion	• Retrograde or absent blood flow during diastole in: -descending aorta OR -coeliac trunk or superior mesenteric artery (SMA) OR -anterior or middle cerebral artery

*A comprehensive echocardiographic assessment should be performed to rule out any underlying congenital heart defect or pulmonary hypertension and delineate orientation of arch (left or right sidedness) before any intervention to close the PDA*.

A large PDA with some or all the signs of hemodynamic significance can be seen in a well infant needing no or minimal ventilatory support, and it may not be clinically significant. There is high rate of spontaneous closure of PDA, even in ELGAN infants, if they are left alone (35, 36). On the other hand, a PDA with similar parameters on echocardiography may be associated with significant hemodynamic instability and co-morbidities (5, 6). The debate remains whether well asymptomatic infants with echocardiographic parameters suggestive of large or hemodynamically significant should be treated or not (37–40).

In the last few years, there has been move toward conservative “watchful” approach in infants with no significant clinical signs even when there are echocardiographic parameters suggestive of large hsPDA (9). The published evidence does not support treating an infant with an echocardiographic parameters suggestive of hsPDA without any significant clinical signs of PDA. Large trials have failed to demonstrate any long term benefit (such as death or BPD) from PDA treatment, although they demonstrated short term benefits in terms of decreasing incidence of large intraventricular hemorrhage or pulmonary hemorrhage (39). However, these trials were underpowered to study the long term effects and even more importantly most of these trials used very limited echocardiography dataset to enroll infants (39–43). Hence, in the absence of evidence the debate of whether to treat or not continues and in fact when to treat, how to treat and who to treat remains controversial. There is no consensus on management of PDA in the extremely preterm infants and even there is no consensus on the definition of the commonly used term “hsPDA.”

Most clinicians would agree that ductus arteriosus with echocardiographic parameters suggestive of large PDA associated with pulmonary over-circulation and systemic hypoperfusion should be regarded as hsPDA (44). Infants with clinical signs of heart failure and echocardiographic signs of a hsPDA would benefit from treatment. They should be treated with diuretic therapy to offload the heart (decrease preload) and they may benefit from fluid restriction, especially if they are on fluid therapy. This approach would help in cardiovascular stabilization prior to definitive intervention (medical, surgical, or transcatheter approach). Whether all hsPDA should be treated or not, and if treated how best they should be treated, remains debatable. In authors' opinion, infants with echocardiographic signs of hsPDA should be either treated or carefully monitored with low threshold for intervention if clinical condition worsens or does not improve.

The treatment intervention varies from no intervention (“wait and watchful” policy) to treating all PDAs diagnosed on echocardiography. “One size may not fit all”—this truly applies on managing PDA in ELGAN infants with huge variation in clinical course and associated co-morbidities. The lack of evidence on how to best evaluate the PDA in these infants probably results in lack of consensus among the scientific bodies, or even among the clinicians within the same unit (45). The intervention options are: no intervention (conservative management), pharmacological treatment (prophylactic medication, early targeted intervention and treatment of symptomatic infants), transcatheter closure and surgical ligation of PDA.

Studies of prophylactic treatments using indomethacin show reduced rates acute pulmonary hemorrhage, intracranial hemorrhage, and surgical ligation (39). It does however expose large proportion of infants to indomethacin who do not need treatment because of spontaneous closure. Studies on prophylactic use of ibuprofen failed to show the benefits reported on using indomethacin (46). Many ongoing studies (BabyOSCAR trial, French TRIOCAPI trial, Dutch BeNEDuCTUS trial, and Australian U-PDA trial focused on the short term and long term outcomes of early targeted treatment approaches ^2^.

With advances in technologies and expertise, PDA can be closed via transcatheter route with faster recovery and avoiding the risk of complications from surgery. This can now be safely done in ELGAN infants weighing over 700 g (47, 48). A detailed discussion about the evidence for various treatment options is beyond the scope of this review article.

**Figure 11:**
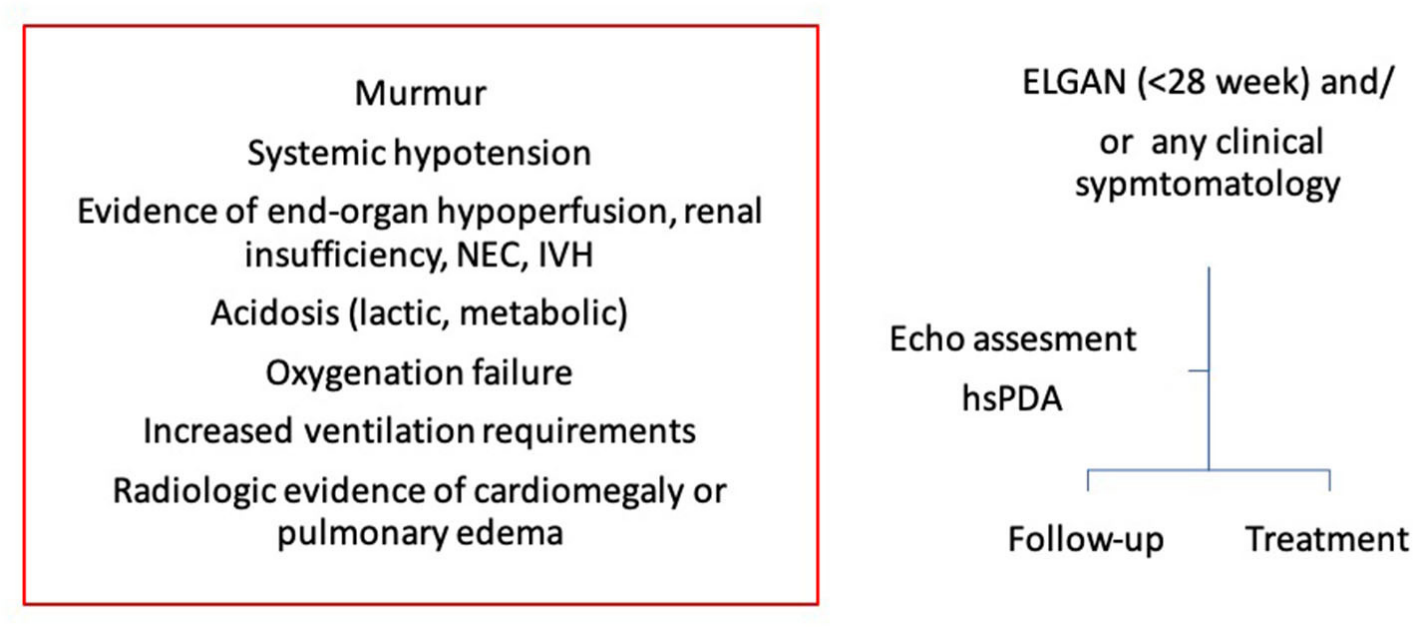
A simple algorithm to clinical decision making in extremely low gestational age newborn infants (ELGAN). Clinical decision whether to treat or follow up should be made based upon both clinical and echocardiography assessment. NEC, necrotizing enterocolitis; IVH, intraventricular hemorrhage; ELGAN, extremely low gestational age newborn infants; hsPDA, hemodynamically significant patent ductus arteriosus.

The authors recommend a comprehensive assessment of PDA and its hemodynamic significance before making any clinical decision to treat or not and the clinical decision making should be made in clinical context of the individual infant. A simple algorithm to clinical decision making based upon clinical and echocardiographic assessment, whether to treat or follow up, has been summarized in Figure 11. Moreover, this can be dynamic decision, especially in infants with conservative management and serial assessment on neonatologist performed echocardiography can help in making timely individualized decision making.

## Conclusion

While the best treatment option to treat the PDA in the extremely low gestational age newborn infants remains debatable and lacks consensus at this stage, there has been significant progress in assessing hemodynamic significance of the ductal shunt. Before making a clinical decision to treat a PDA or not, all infants should have a comprehensive structural assessment to rule out any underlying CHD and a meticulous hemodynamic evaluation to assess the impact of PDA shunt on the pulmonary over-circulation and systemic hypoperfusion. In absence of clear evidence, the clinical decision should be individualized based upon clinical concerns and echocardiographic assessment. While in some infants a careful “wait and watchful” strategy may be the best option, other infants may need early intervention to minimize the co-morbidities. There is an urgent need of studying the long term outcomes in the sub-set of infants with hsPDA identified on meticulous echocardiographic assessment.

## Data Availability Statement

The original contributions presented in the study are included in the article/supplementary materials, further inquiries can be directed to the corresponding author.

## Author Contributions

YS conceptualized the idea and prepared initial manuscript including all images. AF, OE, and BA edited the manuscript and helped in finalizing the manuscript. All authors contributed to the article and approved the submitted version.

## Conflict of Interest

The authors declare that the research was conducted in the absence of any commercial or financial relationships that could be construed as a potential conflict of interest.

## Footnotes

^1^Available online at: https://www.uptodate.com/contents/patent-ductus-arteriosus-in-preterm-infants-pathophysiology-clinical-manifestations-and-diagnosis/abstract/55.

^2^Available online at: https://clinicaltrials.gov/ct2/results?term=pDA&Search=Apply&recrs=a&age.
